# Sulfur rich electron donors – formation of singlet *versus* triplet radical ion pair states featuring different lifetimes in the same conjugate†
†Electronic supplementary information (ESI) available: Emission spectra; transient absorption spectra; pulse radiolysis spectra; HPLC spectra; description of the synthesis. See DOI: 10.1039/c6sc03207a



**DOI:** 10.1039/c6sc03207a

**Published:** 2016-10-05

**Authors:** Avishek Saha, Muqing Chen, Marcus Lederer, Axel Kahnt, Xing Lu, Dirk M. Guldi

**Affiliations:** a Department of Chemistry and Pharmacy , Interdisciplinary Center for Molecular Materials (ICMM) , Friedrich-Alexander University of Erlangen-Nürnberg , Egerlandstr. 3 , 91058 Erlangen , Germany . Email: dirk.guldi@fau.de; b State Key Laboratory of Materials Processing , School of Material Science and Engineering , Huazhong University of Science and Technology , 1037 Luoyu Road , 430074 Wuhan , China . Email: lux@hust.edu.cn

## Abstract

An unprecedented family of novel electron-donor acceptor conjugates based on fullerenes as electron acceptors, on one hand, and triphenyl amines as electron donors, on the other hand, have been synthesized and characterized in a variety of solvents using steady state absorption/emission as well as transient absorption spectroscopy. These are unprecedented in terms of their outcome of radical ion pair formation, that is, the singlet *versus* triplet excited state. This was corroborated by femto/nanosecond pump probe experiments and by molecular orbital calculations. Not only has the donor strength of the triphenylamines been systematically altered by appending one or two sulfur rich dithiafulvenes, but the presence of the latter changed the nature of the radical ion pair state. Importantly, depending on the excitation wavelength, that is, either where the fullerenes or where the triphenylamines absorb, short-lived or long-lived radical ion pair states, respectively, are formed. The short-lived component with a lifetime as short as 6 ps has singlet character and stems from a fullerene singlet excited state precursor. In contrast, the long-lived component has a lifetime of up to 130 ns in THF, has triplet character, and evolves from a triplet excited state precursor. Key in forming more than three orders of magnitude longer lived radical ion pair states is the presence of sulfur atoms, which enhance spin–orbit coupling and, in turn, intersystem crossing. Independent confirmation for the singlet *versus* triplet character came from temperature dependent measurements with a focus on the radical ion pair state lifetimes. Here, activation barriers of 2.4 and 10.0 kJ mol^–1^ for the singlet and triplet radical ion pair state, respectively, were established.

## Abstract 2

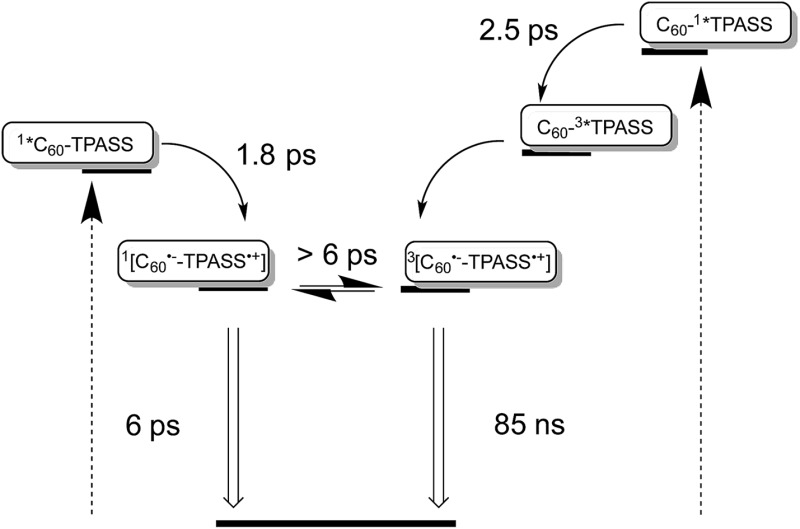
An unprecedented family of novel electron-donor acceptor conjugates based on fullerenes have been synthesized and characterized in a variety of solvents.

## Introduction

Natural photosynthesis is one of the essential processes that runs organic life on earth.^1^ It operates based on the mutual interplay between light harvesting, energy transfer, electron transfer, and catalysis. Inspired by this, a plethora of covalent and non-covalent electron donor–acceptor systems, in which electron donating and electron accepting building blocks are integrated, have been synthesized and investigated. For instance, the most commonly employed electron donors range from chlorophylls and carotenoids to porphyrins/phthalocyanines and triphenylamines. As far as electron acceptors are concerned, perylenebisdiimides, napthalenediimides, endohedral fullerenes, and empty fullerenes should be mentioned. In the resulting systems, particular focus has been placed on fundamental aspects of electron transfer.

In the context of electron acceptors, fullerenes were extensively studied in electron donor–acceptor systems. This is mainly due to their unique π-electronic nature, their marked excited-state electronic properties, and their low reorganization energy in electron transfer reactions.^2–8^ A myriad of possible electron donors has been investigated as counterpart to the electron-accepting fullerene ranging from organic entities like, for example, tetrathiafulvalenes, to complexes based on porphyrins *etc.* ^6,9–16^ What renders the resulting electron donor–acceptor systems most appealing is the possibility to adjust their structure and, in turn, to fine-tune their physicochemical features such as quantum yields and lifetimes of charge separation.^17,18^

Among the electron donors, triphenylamines and their derivatives stand out owing to their good charge transport ability. As a matter of fact, they have been widely used as hole injectors or as active materials in organic electronic devices.^19–23^

Photophysical assays with a system where C_60_ is covalently linked to a TPA, revealed that the photoinduced charge separation process predominantly produces a TPA˙^+^–C_60_˙^+^ radical ion pair state in polar solvents *via* 
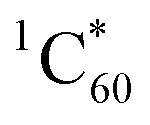
 excitation.^6^ Later, in the analogous TPA–Sc_3_N@C_80_, in which TPA is connected *via* the nitrogen, a significant improved thermal stability resulted in longer lived radical ion pair states when compared to the corresponding 2-substituted conjugate.^24^ This led us to consider a different strategy to alter the lifetime of the radical ion pair state, namely TPA modification. For example, dithiafulvenes, derivatives of tetrathiafulvalene, are known for their high electron density and hole mobility.^25,26^ As such, they bear great potential for modifying TPA and, in turn, for extending the lifetime of the radical ion pair state.

Like in photosynthesis, the lifetime of the radical ion pair state is crucial to link energy conversion to efficient energy storage. To this end, spatially separating electron donors and acceptors is a promising approach, while electronically decoupling them is yet another.^27–30^ Products of both approaches are long-lived radical ion pair states of singlet or triplet nature. A less frequently explored approach employs the conversion of the singlet radical ion pair state character to the corresponding triplet manifold.^31,32^ Due to the needed spin-conversion, which proceeds *via* hyperfine induced singlet–triplet mixing, starting with a triplet excited state precursor in electron donor–acceptor systems enables the efficient formation of long-lived, triplet radical ion pairs. Next to transition metals, whose internal heavy-atom effects facilitate the rapid formation of triplet excited state precursors, second-order vibronic spin–orbit coupling in sulfur-containing building blocks provides a similar means.^33,34^ To the best of our knowledge, however, the formation of singlet *versus* triplet radical ion pairs in the same electron donor–acceptor conjugate is unprecedented. Especially, the simple variation of the excitation wavelength has not been shown to be linked to such a different electron transfer outcome.

**Scheme 1:**
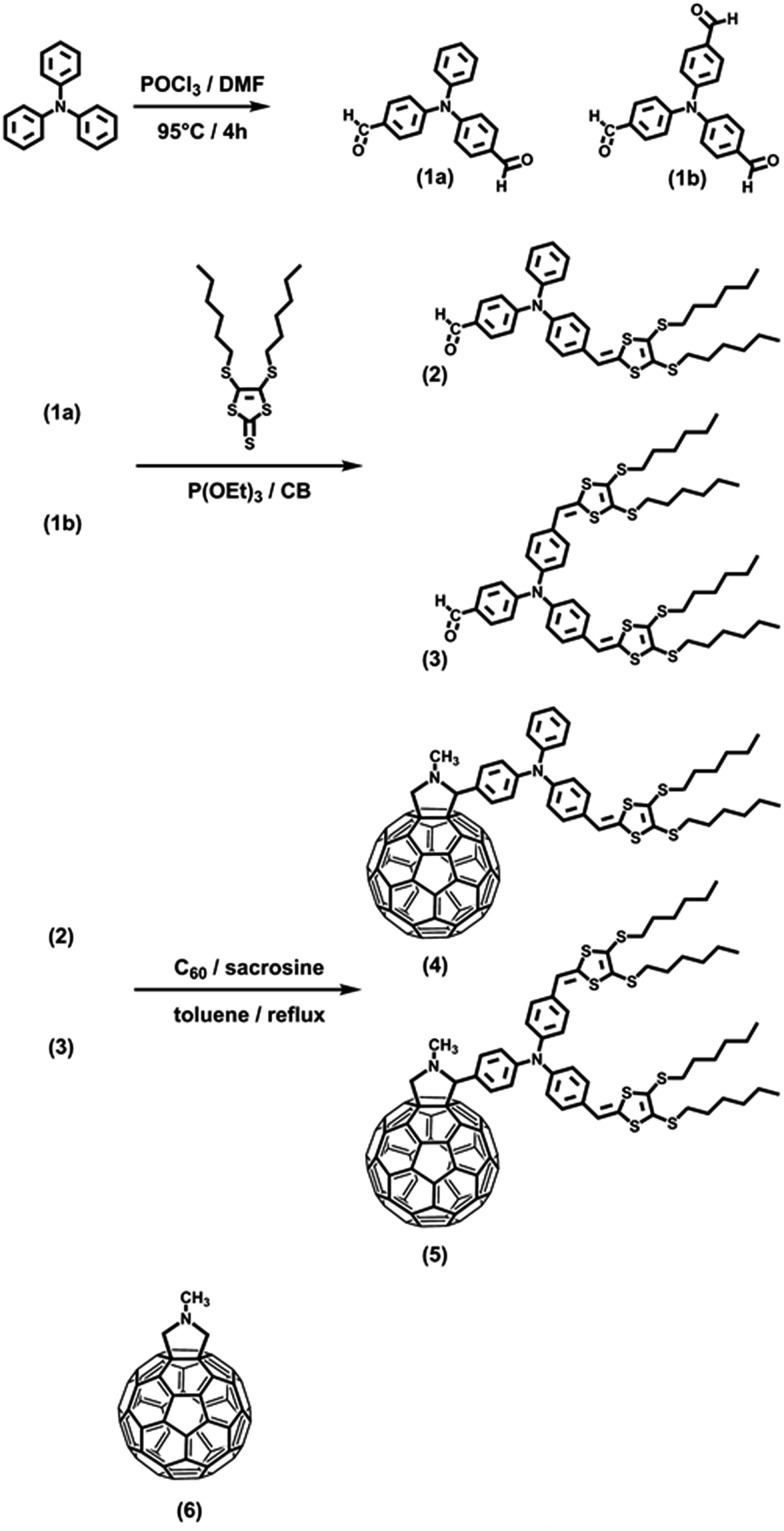
Synthesis of **4** and **5** as well as reference **6**.

In this regard, we have synthesized two novel TPA–C_60_ electron donor–acceptor conjugates **4** and **5** bearing one or two dithiafulvenes by means of the 1,3-dipolar cycloaddition of azomethine ylides – Scheme 1. We have investigated their photophysical properties with a particular focus on photoinduced electron transfer processes, which resulted in radical ion pair states followed by deactivation to the ground state. Importantly, our investigations have enabled for the first time the selective and direct formation of either singlet or triplet radical ion pair states in the same electron donor–acceptor conjugates. To this end, photoexcitation of either the electron accepting fullerenes or the electron donating triphenylamines/dithiafulvenes provide the means for activating different charge separation and recombination pathways. As such, we believe that our work will trend-set the field of electron donor–acceptor design by means of adapting “triplet” precursors.

## Results and discussion

### Ground state characterization

**Fig. 1:**
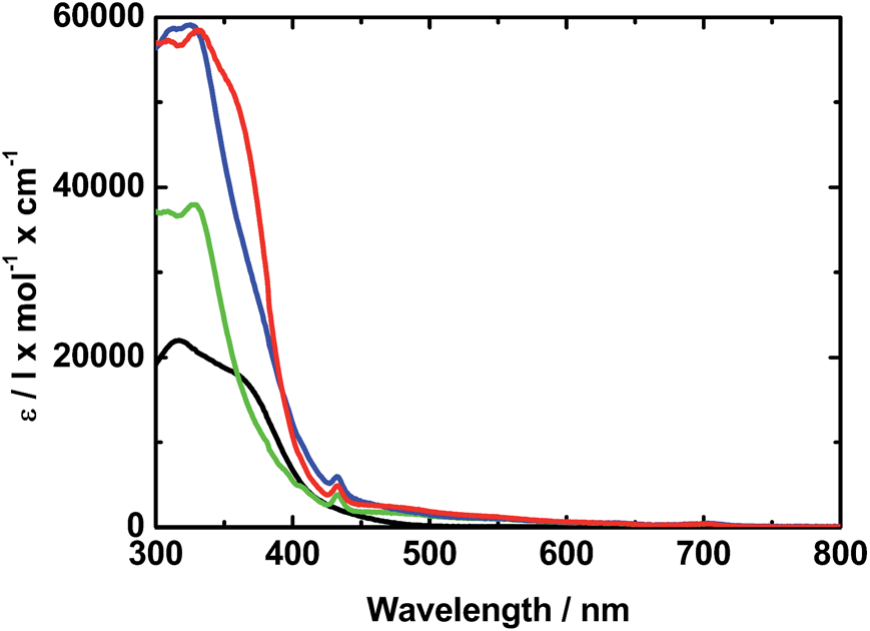
Absorption spectra of **2** (black), **6** (green), and **4** (red) in toluene. The blue line depicts the sum of the absorption spectra of **2** and **6**.

In the absorption spectrum of TPA–dithiafulvene **2**, strong absorptions in the near-UV region of the optical spectrum in the form of a maximum at 315 nm and a shoulder around 370 nm are noted. *N*-Methylfulleropyrrolidine **6** gives rise to absorptions in the near-UV and blue region of the optical spectrum with a dominating maximum at 325 nm. Regarding TPA–dithiafulvene–C_60_ **4**, the ground state absorption is best described as the superimposition of the absorptions of **2** and **7**. In addition, a weak absorption tail that extends into the violet/blue region of the solar spectrum emerges. This is explained by the enhanced conjugated π-system in **4** – see Fig. 1.

**Fig. 2:**
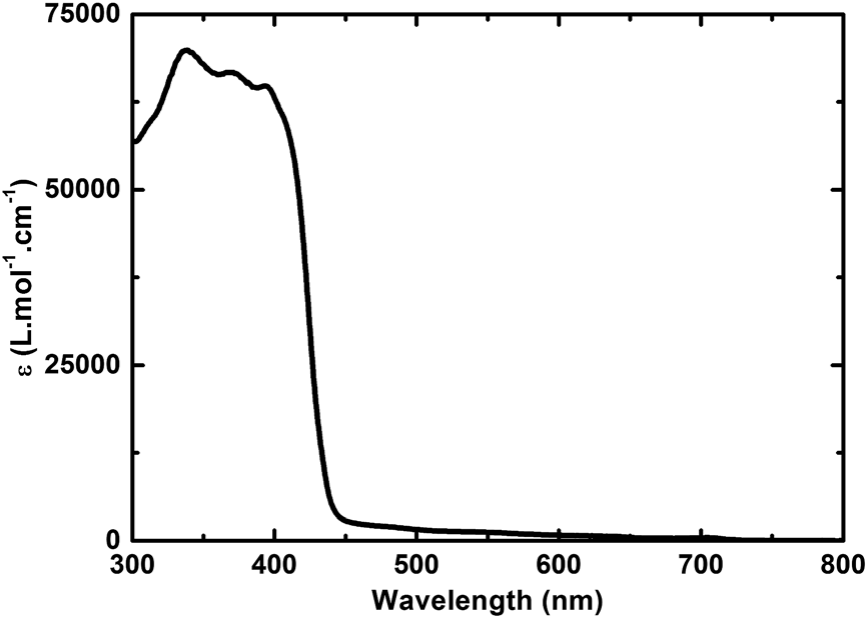
Absorption spectrum of **5** in toluene.

Turning to **3**, absorptions are seen in the UV region of the solar spectrum with a maximum at 317 nm accompanied by a shoulder-like maximum at around 360 nm. For **5**, optical absorptions are located within the same region of the optical spectrum as observed for **3**, with maxima at 338, 369, and 395 nm. Similar to **4**, the absorption of **5** appears as the superimposition of the absorption spectra of **3** and **6** including an additional red shift by about 40 nm of the absorption characteristics located at 400 nm – Fig. 2 and S2.†

### Pulse radiolysis

In order to characterize the absorption spectra of **2**˙^+^ and **3**˙^+^, pulse radiolysis experiments under oxidizing conditions were performed. **2** and **3** were dissolved in 1-chlorobutane (BuCl), saturated with N_2_ and irradiated with short high energy electron pulses. These conditions lead to the formation of BuCl˙^+^, which is known as a strong oxidizing agent.^35,36^ The ability of BuCl˙^+^ oxidizing aromatic amines *via* free electron transfer is well established in the literature.^37,38^ So it is safe to assume that **2** and **3** are oxidized like TPA – described in the literature as forming **2**˙^+^ and **3**˙^+^, respectively.^37^

The pulse radiolysis spectrum of solutions of **2** in BuCl shows, after the electron pulse, the typical transient absorption of BuCl˙^+^ with a characteristic transient absorption maximum around 520 nm (not shown).^35,36,39^ This transient absorption decays rapidly giving rise to a new set of transient absorptions, one covering the visible part of the optical spectrum and one in the NIR – see Fig. S10.† These transient absorption bands are attributed to the transient absorption of **2**˙^+^.

Regarding the reaction of **3** with BuCl˙^+^, again directly after the electron pulse, the characteristic transient absorption features of the BuCl˙^+^ were observed, which rapidly decay, giving rise to new transient absorption bands maximizing at 580 and 1100 nm – see Fig. S6† – belonging to the transient absorption of **3**˙^+^.

### Molecular orbital calculations

**Fig. 3:**
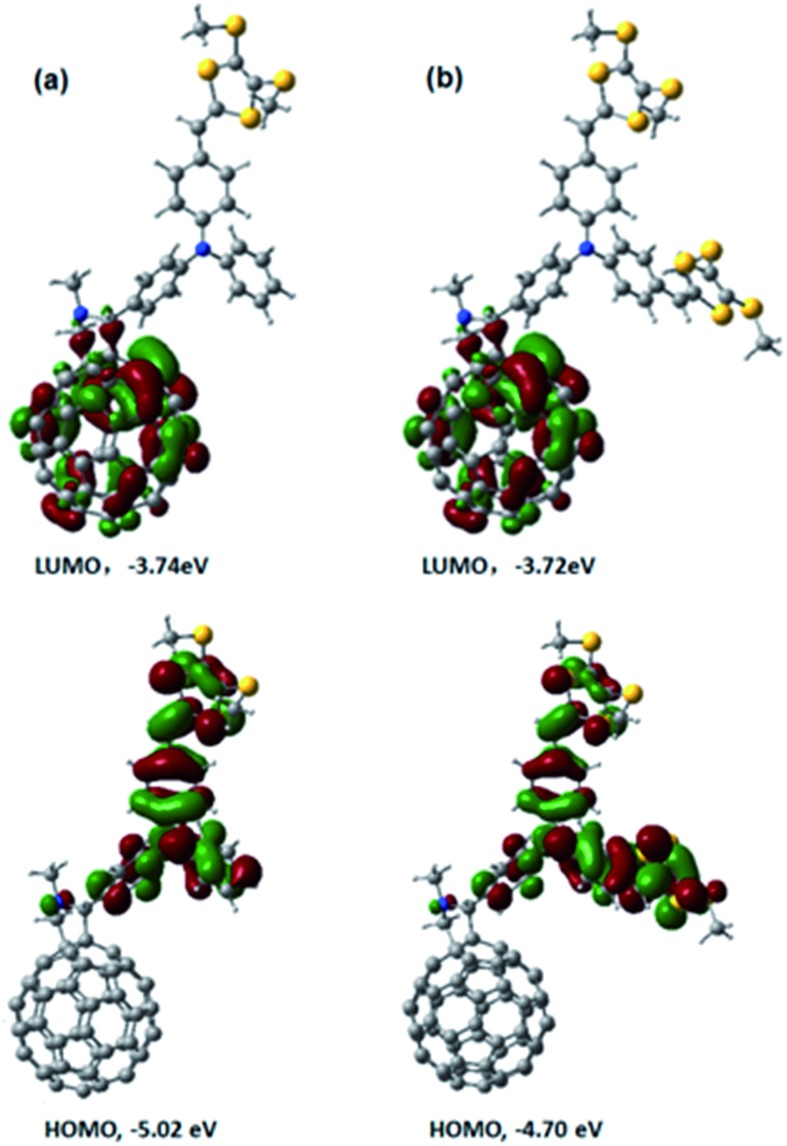
Optimized structures and electron densities of LUMOs and HOMOs of (a) **4** and (b) **5** calculated at the B3LYP/3-21G level of theory.

Next, we performed computational studies to shed light onto the geometrical and electronic features of **4**, **5**, and C_60_–TPA lacking dithiafulvenes. All of the structures were optimized at the B3LYP/3-21G level using the Gaussian 09 program package. Fig. 3 shows the spatial electron densities of the highest occupied molecular orbital (HOMO) and the lowest unoccupied molecular orbital (LUMO) for **4**, **5**, and C_60_–TPA. First of all, the electron density of the HOMO in C_60_–TPA was found to be located on the aromatic amine moiety, while the LUMO is localized on the C_60_ spheroid. These preliminary calculations suggest that the anion radical will be localized on the C_60_ spheroid, while the radical cation will be localized on the aromatic amine moiety in case of a charge separated state, which is in line with the work of O. Ito *et al.* ^6^ Secondly, for **4** and **5**, the corresponding LUMOs are mainly localized on the fullerene, while the spatial electron densities of the HOMO are extended to the side chains, which bear the dithiafulvene ring. In summary, functionalization of the TPA moiety tunes the energies of the HOMOs (better electron donating ability) while the energies of the LUMOs remain essentially unchanged – see Fig. 3.

### Excited state characterization

The first insight into possible electron donor acceptor interactions came from fluorescence assays. **2**, for example, exhibits a strong emission between 400 and 650 nm with a maximum at 435 nm – Fig. S3 in the ESI† – and an emission quantum yield of 0.007 in toluene. When turning to **4**, the TPA–dithiafulvene centered emission is completely quenched in toluene and in THF. For TPA–bis-dithiafulvene **3**, the strong emission between 400 and 500 nm maximizes at 425 nm – Fig. S3.† A value of 0.015 was obtained for the emission quantum yield in toluene. For **5**, the TPA–bis-dithiafulvene based emission is fully quenched in toluene and THF.

As a complement, we investigated C_60_ based fluorescence. Here, in order to excite C_60_ exclusively, 470 nm was selected as excitation wavelength. *N*-Methylfulleropyrrolidine **6** was used as reference component with a reported fluorescence quantum yield of 6.0 × 10^–4^ in toluene.^40^ **4**, **5**, and **6** all show typical *N*-methylfulleropyrrolidine centered emissions^40^ with maxima located at 710 ± 1 nm – Fig. S4.† In toluene, only **5** showed a significant quenching of the C_60_ fluorescence with a quantum yield of 3.2 ± 0.5 × 10^–5^. Turning to THF, the fluorescence of **4** is quenched by an order of magnitude (5.3 ± 0.5 × 10^–5^). In **5**, the fluorescence was quantitatively quenched in THF.

In summary, the complete quenching of the TPA–dithiafulvene and TPA–bis-dithiafulvene fluorescence in **4** and **5** relative to references **2** and **3**, respectively, points to an additional decay mechanism – energy transfer and/or electron transfer. Likewise, quenching of the C_60_ fluorescence in **4**/THF, in **5**/toluene, and in **5**/THF indicates an additional decay process starting from 
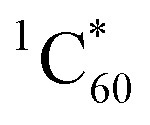
.

Transient absorption spectroscopy based on femtosecond and nanosecond laser photolysis was employed to shed light onto the additional decay processes. In the case of **2** and **3**, the singlet excited states are formed immediately after laser excitation. Both show strong transient absorptions throughout the visible and NIR region of the optical spectrum with maxima at 615 for **2** or 612 nm for **3** – Fig. S5.† The latter decay with a lifetime of ∼2.5 ps and transform into transient absorptions maximizing at either 565 nm (**2**) or 590 nm (**3**). A likely rationale infers formation of the corresponding triplet manifold by means of rapid intersystem crossing due to the presence of sulfur atoms. In both cases, that is, for **2** and **3**, these transient absorptions decay with lifetimes of 370 and 480 ps, respectively, to repopulate the ground state.

**Fig. 4:**
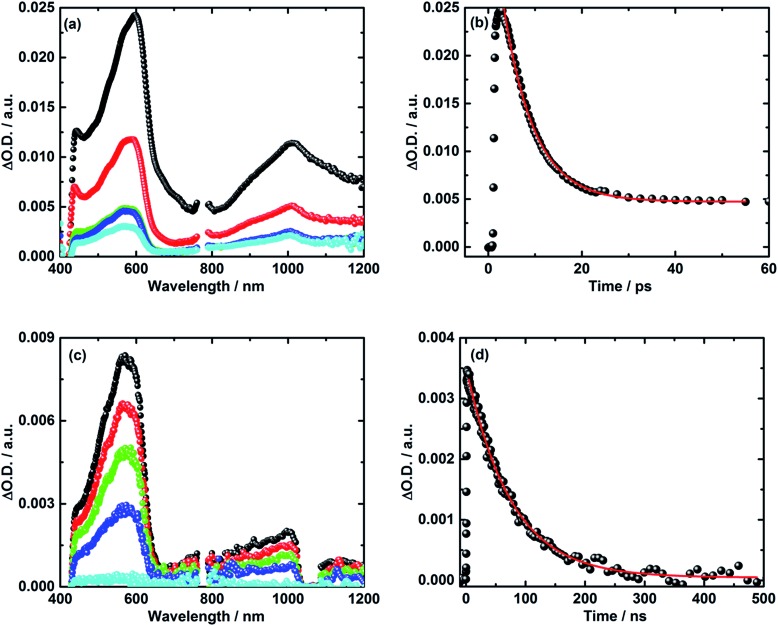
(a) Transient absorption spectra of **5** in argon saturated THF upon fs-laser photolysis (387 nm, 400 nJ per pulse) with time delays of 2 ps (black), 10 ps (red), 100 ps (green), 1000 ps (blue), and 5000 ps (cyan) after the laser pulse. (b) Corresponding time absorption profile (black) and exponential decay fit (red) at 590 nm. (c) Nanosecond transient absorption spectra of **5** in argon saturated THF upon laser photolysis (387 nm, 400 nJ per pulse) with time delays of 5 ns (black), 25 ns (red), 50 ns (green), 100 ns (blue), and 500 ns (cyan) after the laser pulse. (d) Corresponding time absorption profile (black) and exponential decay fit (red) at 590 nm.

Turning to the electron donor–acceptor conjugate **5** upon 387 nm photoexcitation with femtosecond laser pulses in THF, two transient absorption bands appear 3 ps after the laser pulse.^41^ For **5**, the maxima occur around 590 and 1010 nm – see Fig. 4a and b. Notably, the latter absorption is diagnostic for the one electron reduced C_60_ radical anion, whereas the former matches well the transient absorption fingerprint of the one electron oxidized TPA–bis-dithiafulvene radical cation. The latter was determined *via* pulse radiolytic oxidation – Fig. S6.† From this assignment we conclude the successful formation of the TPA–bis-dithiafulvene˙^+^–C_60_˙^–^ radical ion pair state in conjugate **5**. Taking a closer look at the decay of the TPA–bis-dithiafulvene˙^+^–C_60_˙^–^ radical ion pair state in THF the transient absorption features decay biphasic. In particular, a short-lived component was found along with a long-lived component. For the short lived component a lifetime of 6 ps has been derived while the long lived component decays on a timescale beyond the detection limit of our femtosecond laser photolysis setup of 5500 ps – see Fig. 4.

In order to reveal the identity and the lifetime of the long lived transient, the same experiments were repeated with a nanosecond laser photolysis setup. Upon photoexcitation at 387 nm, the same transient absorption appeared as observed in the femtosecond laser photolysis experiments – *vide infra*. In other words, maxima at 590 and 1010 nm confirm the formation of the TPA–bis-dithiafulvene˙^+^–C_60_˙^–^ radical ion pair state. Next to seeing the short lived component with its 6 ps lifetime, the long lived component was seen to decay with a lifetime of 85 ns – Fig. 4c and d. In Fig. S7,† normalized transient absorption spectra from femtosecond and nanosecond laser pulse measurements are shown to demonstrate the resemblance of transients across the different timescales. Any notable differences, as they are noted in the near-infrared parts of the transient absorption spectra, are based on the different white light sources used for the femto- and nanosecond laser photolysis setups.

Our findings that the radical ion pair state shows two lifetimes and that a 387 nm excitation excites TPA–bis-dithiafulvene as well as C_60_ – Fig. 1 and 2 – lead us to hypothesize that two radical ion pair states are formed. One of them is born upon exciting C_60_, while the other stems from exciting TPA–bis-dithiafulvene. The different radical ion pair state lifetimes are only reasonable when different spin multiplicities, that is, singlet *versus* triplet, are considered. In other words, we postulate the competition of a singlet *versus* triplet radical ion pair state.

It is well documented that photoexcitation of C_60_ leads to its singlet first excited state, for which a lifetime of 1.4 ns is found unless it is quenched.^42^ Please note that the initially populated C_60_ singlet excited state with its characteristic absorption maximum at around 900 nm is invisible due to the rapidly occurring charge separation. Considering that the spin polarization remains constant during the electron transfer, it is reasonable to postulate the formation of a short-lived singlet radical ion pair state, originating from the C_60_ singlet excited state. This leaves the long lived radical ion pair state with a triplet electron spin multiplicity. The only feasible pathway in electron donor–acceptor conjugate **5** towards a triplet radical ion pair state is a triplet excited state precursor. Here, the strong spin orbit coupling induced by sulfur atoms comes into play as it facilitates in **2** and **3** intersystem crossing from the TPA–bis-dithiafulvene and TPA–dithiafulvene singlet first excited states to the corresponding triplet excited states.^43^

The formation of radical ion pair states with different spin multiplicities has been reported by Fukuzumi *et al.* ^44^ A common verification of the triplet spin multiplicity is the quenching of the parent triplet excited state or the triplet radical ion pair state with molecular oxygen. In the current case, this is, however, unfeasible. The triplet quenching by oxygen is based on a bimolecular energy transfer and the lifetimes observed for our compounds are too short to observe reliable and interpretable oxygen effects.

**Fig. 5:**
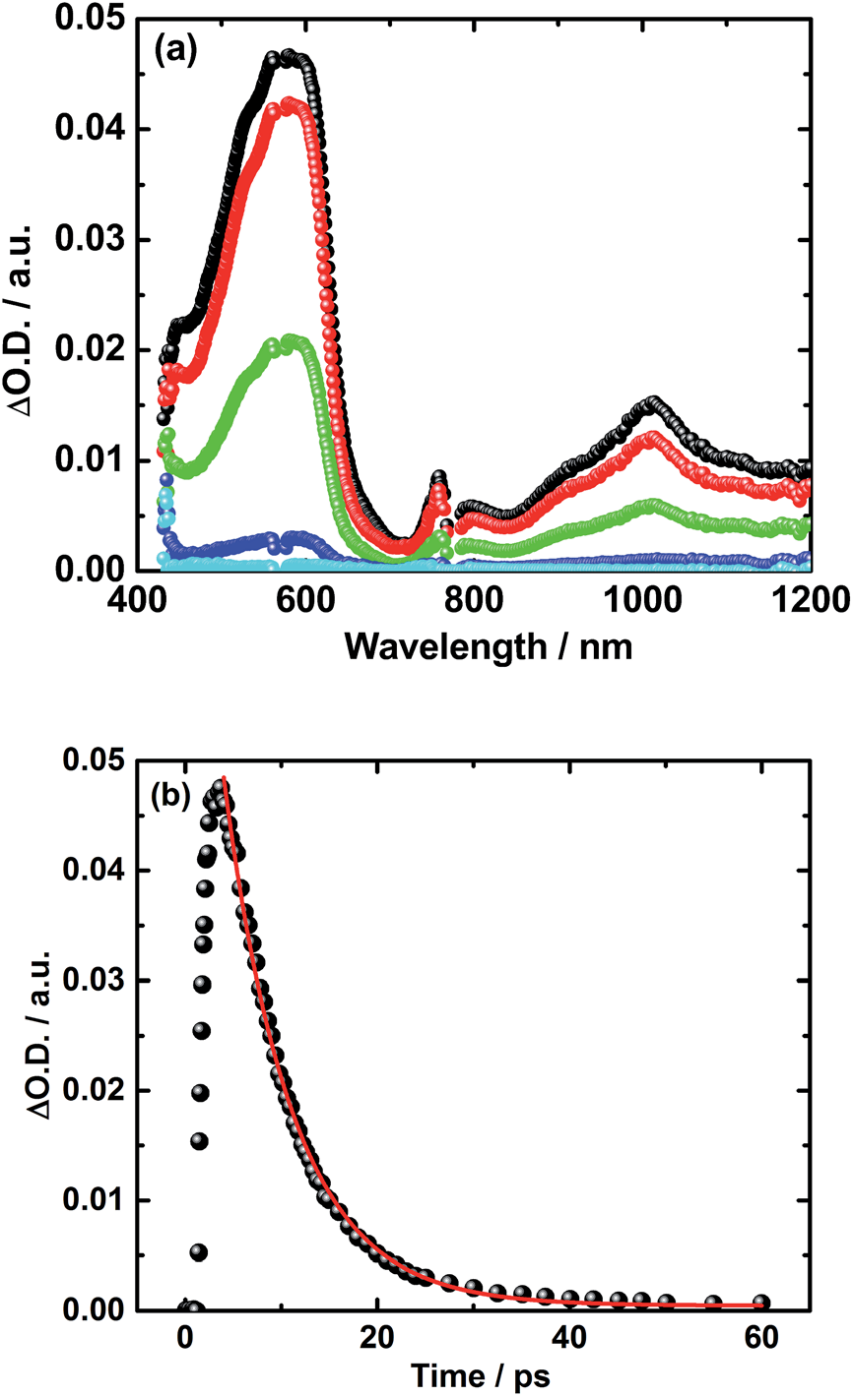
(a) Transient absorption spectra of **5** in argon-saturated THF upon fs-laser photolysis (568 nm, 400 nJ per pulse) with time delays 2 ps (black), 5 ps (red), 10 ps (green), 25 ps (blue), and 100 ps (cyan) after the laser pulse. (b) Corresponding time absorption profile (black) and exponential decay fit (red) at 590 nm.

A different approach to verify our hypothesis is based on taking advantage of the different absorption cross sections. Considering, for example, the absorption spectra of TPA–bis-dithiafulvene **3** – Fig. 2 – and C_60_ reference **7** – Fig. 1, we performed femtosecond transient absorption experiments by exciting solutions of **5** at 568 nm. Only contributions from the C_60_ singlet excited state should be discernable, due to the lack of TPA–bis-dithiafulvene absorption. This is, indeed, the case. Importantly, exciting at 568 nm shows transient absorptions exclusively attributed to the short lived radical ion pair state – Fig. 5 – which is formed with 1.8 ps.

As a complement, we performed femtosecond transient absorption measurements following 420 nm fs-laser excitation. The latter generates both a C_60_ singlet excited state and a TPA–bis-dithiafulvene singlet excited state, similar to those experiments in which 387 nm excitation was employed. Notably, the TPA–bis-dithiafulvene singlet excited state transforms rapidly *via* intersystem crossing into the corresponding triplet manifold. Like in the 387 nm excitation experiments, biphasic decay kinetics are observed. In particular, a short-lived 6 ps component accompanies a long-lived 85 ns component – Fig. S8.† At the 420 nm excitation, the relative C_60_ to TPA–bis-dithiafulvene absorption cross section increases and, in turn, contributions from the C_60_ singlet excited state dominate over those of the TPA–bis-dithiafulvene triplet excited state. In line with the latter, the relative ratio between the short- and long-lived radical ion pair states intensified.

**Fig. 6:**
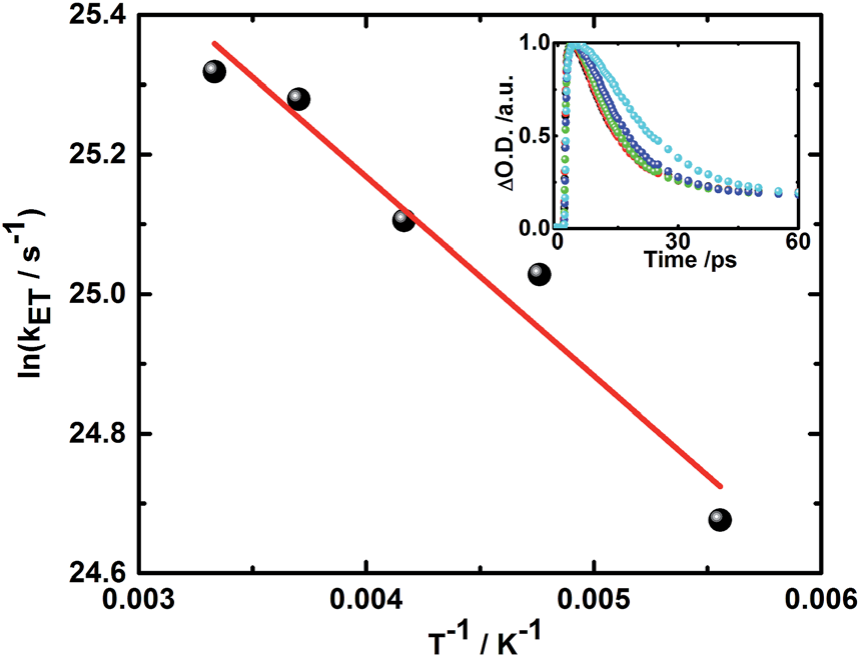
Plot of rate constant for the charge recombination of the singlet radical ion pair state *vs. T* ^–1^ for **5** in 2-methyl THF upon photoexcitation at 387 nm. Linear fit is shown in red. Inset: corresponding time–absorption profiles at 600 nm obtained at 300 K (black), 270 K (red), 240 K (green), 210 K (blue), and 180 K (cyan).

**Fig. 7:**
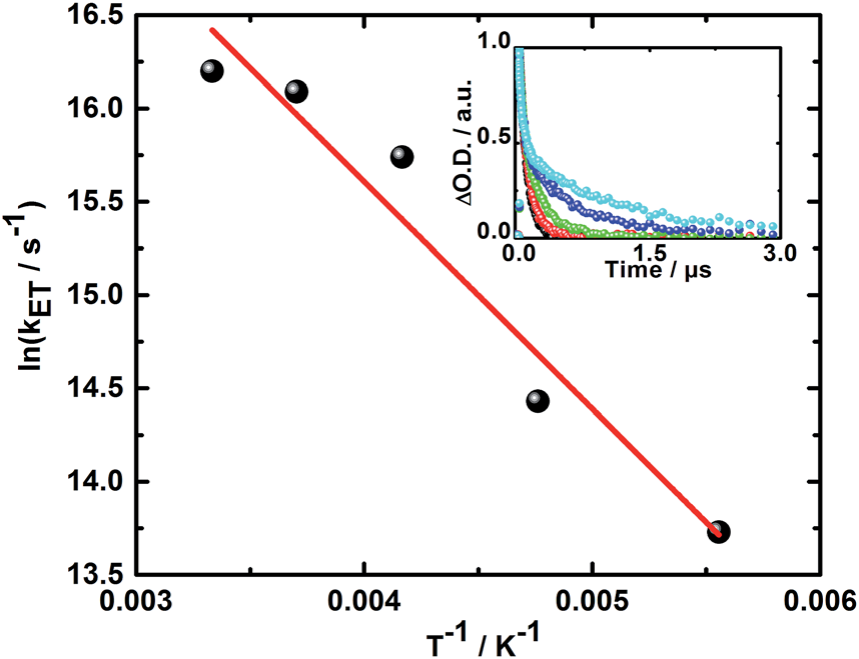
Plot of the rate constant for the charge recombination of the triplet radical ion pair state *vs. T* ^–1^ for **5** in 2-methyl THF upon photoexcitation at 387 nm. Linear fit is shown in red. Inset: corresponding time–absorption profiles at 600 nm obtained at 300 K (black), 270 K (red), 240 K (green), 210 K (blue), and 180 K (cyan).

Additionally, the finding of two different radical ion pair states – one with singlet and one with triplet spin multiplicity – was confirmed in temperature dependent laser photolysis transient absorption measurements with, for example, **5**. When reducing the temperature from 300 to 180 K in 2-methyl-THF the lifetime of the radical ion pair state with singlet spin multiplicity increases from 10 ps (300 K) to 19 ps (180 K) – Fig. 6. From the fit to the Arrhenius equation, a rather small activation barrier for the charge recombination of the singlet radical ion pair state of 2.4 kJ mol^–1^ was obtained. When turning to the temperature dependency of the triplet radical ion pair state of **5** in 2-methyl THF, the lifetime increases from 92 ns (300 K) to 1090 ns (180 K) – Fig. 7. From an Arrhenius analysis, a fairly substantial activation barrier of 10.0 kJ mol^–1^ was derived for the charge recombination of the triplet radical ion pair state.

Both activation barriers, that is, 2.4 and 10.0 kJ mol^–1^ for the charge recombination of the singlet and triplet radical ion pair states, respectively, are well in line with our expectations. In **5**, the activation barrier for the recombination of the singlet radical ion pair state is comparable to RT at room temperature and, in turn, negligible.

In stark contrast, the activation energy for the triplet radical ion pair state is substantially higher due to the presence of parallel electron spins. The latter requires spin flipping as the rate-limiting step in the repopulation of the ground state.

**Fig. 8:**
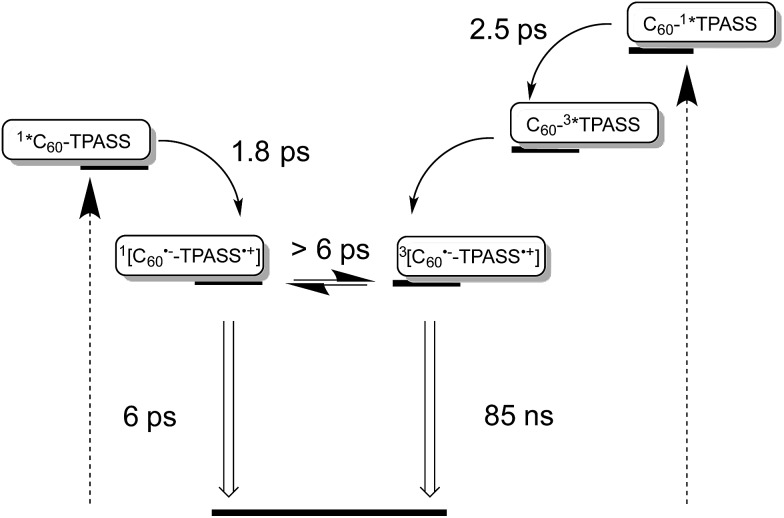
Simplified energy diagrams for **5** in THF illustrating on the left and on the right the different deactivation pathways upon photoexcitation of TPA–bis-dithiafulvene (TPAS) at 387, 420, and 568 nm and C_60_ at 387 and 420 nm, respectively. The energy of the radical ion pair state energy was determined as 1.25 eV for **5** from the one electron reduction of C_60_ as –1.19 V *versus* Fc/Fc^+^ and the one electron oxidation of TPASS as +0.05 V *versus* Fc/Fc^+^ and as 1.25 eV for **4** from the one-electron reduction of C_60_ as –1.14 V *versus* Fc/Fc^+^ and the one-electron oxidation of TPASS as +0.11 V *versus* Fc/Fc^+^.

Taking the aforementioned in concert, it is safe to conclude that formation of a singlet radical ion pair state results from C_60_ photoexcitation in **5**, while a triplet radical ion pair state evolves from TPA–bis-dithiafulvene photoexcitation – Fig. 8.

Turning to the electron donor–acceptor system **4** featuring only one TPA–dithiafulvene, upon laser excitation with 387 nm femtosecond laser pulses evidence for the short-lived and the long-lived radical ion pair state is noted. In particular, the transient absorption bands maximize at 585 and 1010 nm – Fig. S7 and S11.† On one hand, the feature at 1010 nm is a perfect match of the one-electron reduced C_60_ radical anion.^17,45^ On the other hand, the transient absorption in the visible range of the solar spectrum is in sound agreement with the transient absorption of the one-electron oxidized TPA–dithiafulvene radical cation obtained in our pulse radiolysis study – Fig. S9 and S10.† Our finding that the transient absorption matches the transient absorption of the C_60_ radical anion and the TPA–dithiafulvene radical cation strongly suggests the formation of the TPA–dithiafulvene˙^+^–C_60_˙^–^ radical ion pair state. Like in the case of **5**, the radical ion pair state of **4** decays in 387 nm excitation experiments with two lifetimes, namely 6 ps and 130 ns. One is due to singlet spin multiplicity and one is due to triplet spin multiplicity. In the presence of air, the lifetime of the 130 ns is reduced to 31 ns, from which we conclude an activation controlled deactivation of the radical ion pair state.

Considering the similar absorption spectra for **4** and **5**, 387 nm excitation leads to the formation of the C_60_ singlet excited state as well as to the generation of the TPA–dithiafulvene singlet excited state. The latter, however, rapidly transforms *via* intersystem crossing to the TPA–dithiafulvene triplet excited state. Again, we postulate that from the C_60_ singlet excited state the short lived, singlet radical ion pair state evolves, while the TPA–dithiafulvene triplet excited state is the precursor to the long lived, triplet radical ion pair state. Support for this notion came from femtosecond transient absorption measurements exciting at 568 nm. Similar to what was described for **5**, the 568 nm excitation results exclusively in the C_60_ singlet excited state and, in turn, singlet radical ion pair state formation. Importantly, the TPA–dithiafulvene˙^+^–C_60_˙^–^ transient absorptions decay with a 6 ps component. Here, no long lived transient absorption remains on the time scale beyond 10 ps – Fig. S12.†

## Conclusions

In summary, we have observed fast, photoinduced charge separation in a set of two novel electron donor–acceptor conjugates based on fullerenes and sulfur-containing triphenylamines. Importantly, depending on the excitation wavelength; that is, either where the fullerenes or where the triphenylamines absorb; for the first time short-lived or long-lived TPA–dithiafulvene˙^+^–C_60_˙^–^/TPA–bis-dithiafulvene˙^+^–C_60_˙^–^ radical ion pair states, respectively, are formed in the same conjugate. On one hand, the short-lived component with a lifetime as short as 6 ps has singlet spin multiplicity and stems from a fullerene singlet excited state precursor. On the other hand, the long-lived component has a lifetime of up to 130 ns in THF, has triplet spin multiplicity, and evolves from a triplet excited state precursor. Both are energetically nearly degenerate, but the exact determination requires application of a varying external magnetic field. Key in forming a more than three orders of magnitude longer lived triplet radical ion pair states is the presence of sulfur atoms, which enhance spin–orbit coupling and, in turn, render intersystem crossing of TPA–dithiafulvene/TPA–bis-dithiafulvene in the excited state fast with a lifetime of about 2.5 ps. The change in spin multiplicity of the radical ion pair state is likely to be slower. At room temperature, upper and lower limits are given by the radical ion pair states of 6 ps and 130 ns, respectively. Notably, the interconversion between the singlet and triplet radical ion pair states is characterized by magnetic field strength dependence. The physical process, which allows for such detailed analysis, involves Zeeman splitting of the *T*_0_ state by the magnetic field. It can be modulated by the strength of the magnetic field, which is a path that we are currently exploring in our laboratory. Regardless of the respective spin multiplicity, both radical ion pair states decay directly to the ground state without forming a triplet excited state. In other words, one state decays in a spin-allowed and fast manner, while the other one decays in a spin-forbidden and slow manner. Independent confirmation for the singlet *versus* triplet character came from temperature dependent measurements with a focus on the radical ion pair state lifetimes. Here, activation barriers of 2.4 and 10.0 kJ mol^–1^ for the singlet and triplet radical ion pair state, respectively, were established. Our results document the incentives of a “triplet” precursor and, as such, will pave the way towards new electron donor–acceptor materials for photovoltaics and photocatalysis.

## Experimental

### General methods

All reactions were performed under an argon atmosphere. The ^1^H and ^13^C NMR chemical shifts are given relative to tetramethylsilane (TMS). All chemicals were purchased and used as-received unless otherwise noted. Triphenylamine (TPA) was purchased from Aldrich Chemical Co. 4,5-Bis(hexylthio)-1,3-dithiole-2-thione was prepared according to the reported procedures.^46^

### Synthesis

The details of the syntheses of **1a**, **1b**, **2**, **3**, **4**, and **5** as well as their characterizations are provided in the ESI.†

### Photophysical studies

Steady-state UV/Vis absorption spectra were measured on PerkinElmer Lambda 35 and PerkinElmer Lambda 2 two-beam spectrophotometers. Steady-state emission spectra were recorded with a FluoroMax P spectrometer (Horiba Jobin Yvon). The experiments were performed at room temperature. Emission quantum yields for the TPA-derivative based emission were determined by the comparative method using 9,10-di-phenylanthracene as references with a fluorescence quantum yield of 0.9 in cyclohexane.^47^

Femtosecond transient absorption laser photolysis measurements were performed with an output from a Ti/sapphire laser system (CPA2110, Clark-MXR Inc.): 775 nm, 1 kHz, and 150 fs FWHM pulses. The excitation wavelength was either generated by second harmonic generation (387 nm) or using a NOPA (NOPA Plus – Clark MXR) (420 and 568 nm). For all excitation wavelengths, pulse widths of <150 fs and energies of 400 nJ per pulse were selected. The transient absorption measurements were performed with a HELIOS (Ultrafast Systems LLC) transient absorption spectrometer.

Nanosecond transient absorption measurements were performed with an EOS transient absorption spectrometer (Ultrafast Systems LLC). The pump pulses were generated from the amplified Ti:sapphire laser system described above. The probe pulse (2 kHz, 0.5 ns pulse width), which was generated in a 1064 nm pumped photonic fiber, was synchronized with the femtosecond amplifiers.

Conventional nanosecond laser flash photolysis transient absorption measurements were carried out with the output from the third harmonic (355 nm, 10 mJ) of a Nd/YAG laser (Brilliant B, Quantel). The optical detection is based on a pulsed (pulser MSP 05 – Müller Elektronik Optik) xenon lamp (XBO 450, Osram), a monochromator (Spectra Pro 2300i, Acton Research), a fast InGaAs photodiode (Nano 5, Coherent) with 300 MHz amplification, and a 1 GHz digital oscilloscope (WavePro7100, LeCroy).

### Pulse radiolysis

The samples were dissolved in 1-chlorobutane, saturated with N_2_ and irradiated with high energy electron pulses (1 MeV, 15 ns duration) by a pulse transformer type electron accelerator (Elit – Institute of Nuclear Physics, Novosibirsk, Russia). The dose delivered per pulse was measured by electron dosimetry.^48^ Doses of 100 Gy were employed. The optical detection of the transients was carried out with a detection system consisting of a pulsed (pulser MSP 05 – Müller Elektronik Optik) xenon lamp (XBO 450, Osram), a SpectraPro 500 monochromator (Acton Research Corporation), a R 9220 photomultiplier (Hamamatsu Photonics), and a 500 MHz digitizing oscilloscope (TDS 640, Tektronix).

### Quantum chemical calculations

Quantum chemical calculations were performed based on the DFT method using the B3LYP/3-21G functional as implemented in the Gaussian 09 program package.^49^

### Electrochemical measurements

Cyclic voltammetry was performed by using a FRA **2** μ Autolab type III (METROHM) potentiostat, in a conventional three-electrode electrochemical cell. A glass carbon disk (1 mm diameter) was used as the working electrode, a Pt wire electrode served as the counter electrode, and an Ag wire was used as the quasi reference electrode. Typically, 100 μM solutions of **4** and **5** were prepared in dichloromethane while 0.1 M (*n*-Bu)_4_NPF_6_ was used as the supporting electrolyte. All potentials were referenced to ferrocenium/ferrocene (Fc^+^/Fc). Measurements were performed using a 50 mV s^–1^ scan rate after purging with argon at ambient temperature.
