# Supplementary material for: Sulfur rich electron donors – formation of singlet versus triplet radical ion pair states featuring different lifetimes in the same conjugate
Source: Chem Sci. 2016 Oct 5;8(2):1360–8. doi: 10.1039/c6sc03207a (PMC5361866; doi:10.1039/c6sc03207a)
Supplement: Supplementary file 1 [file SC-008-C6SC03207A-s001.pdf]

Electronic Supplementary Material (ESI) for Chemical Science.
This journal is © The Royal Society of Chemistry 2016

Electronic Supplementary Information

Sulfur Rich Electron Donors – Formation of Singlet versus Triplet Radical Ion Pair States Featuring Different Lifetimes in the Same Conjugate

Avishek Saha,[‡a] Muqing Chen,[‡b] Marcus Lederer,[a] Axel Kahnt,[a] Xing Lu,[*b] and Dirk M. Guldi[*a]

[a]Department of Chemistry and Pharmacy & Interdisciplinary Center for Molecular Materials (ICMM), Friedrich-Alexander University of Erlangen-Nürnberg, Egerlandstr. 3, 91058 Erlangen, Germany

[b]State Key Laboratory of Materials Processing & School of Material Science and Engineering, Huazhong University of Science and Technology, 1037 Luoyu Road, 430074 Wuhan, China

Table of Content

Synthesis of **1a**, **1b**, and **2** — **page S2**

Synthesis of **3**, **4**, and **5** — **page S3**

Figure S1. HPLC profiles of **4** and **5**. — **page S4**

Figure S2. Absorption spectrum of **3**. — **page S5**

Figure S3. Emission spectra of **2** and **3**. — **page S6**

Figure S4. Emission spectra of **4**, **5**, and **6**. — **page S7**

Figure S5. Transient absorption spectra and time absorption profile of **2** and **3** in THF, 387 nm excitation. — **page S8**

Figure S6. Pulse radiolysis spectrum of **3**. — **page S9**

Figure S7. Normalized transient absoprion spectra of **5** in THF. — **page S10**

Figure S8. Transient absorption spectra and time absorption profile of **5** in THF, 420 nm excitation. — **page S11**

Figure S9. Transient absorption spectra of **4** in THF, 387 nm excitation. — **page S12**

Figure S10. Pulse radiolysis spectrum of **2**. — **page S13**

Figure S11. Transient absorption spectra of **4** in THF, 355 nm excitation. — **page S14**

Figure S12. Transient absorption spectra of **4** in THF, 568 nm excitation. — **page S15**

The synthesis of 1a

The $POCl_3$ (31.2 g, 204 mmol) was added dropwise to the DMF (14.9g, 204 mmol) in a three-necked flask under argon atmosphere, and the reaction system was stirred at 0 °C for 1h. Triphenylamine (10 g, 40.8 mmol) then was added to the mixture, and the reaction temperature was heated to 80 °C overnight. After cooling, the solution was poured into ice water (1000 mL) and the resulting mixture was neutralized with NaOH aqueous solution, the precipitate was obtained by vacuum filtration and dried by vacuum. The precipitate was resolved by acetone, filtered and purified by flash column chromatography (petroleum / ethyl acetate = 6/1 (v/v) as the eluent) to get yellow solid of **1a** Yield 63.5 %. ^{1}H NMR (600 MHz, Acetone) δ 9.94 (s, 2H), 7.87 (dd, *J* = 7.5, 1.2 Hz, 4H), 7.53 – 7.43 (m, 2H), 7.37 – 7.30 (m, 1H), 7.30 – 7.17 (m, 6H). ^{13}C NMR (151 MHz, Acetone) δ 190.20, 151.95, 145.75, 131.66, 131.02, 130.21, 127.19, 126.22, 122.71. MS (MALD-TOF) :m/z: 300.126.

The synthesis of 1b

*The synthesized procedure of **1b** was as follow as the **1a,*** $POCl_3$ (18.7 g, 122.5 mmol), DMF (8.9 g, 122.5 mmol), **1a** (6 g, 19.9 mmol). The resulting mixture was heated at 80 ^{o}C overnight. After cooling, the solution was poured into ice water (1000 mL) and the resulting mixture was neutralized with NaOH aqueous solution, the precipitate was obtained by vacuum filtration and dried by vacuum. The precipitate was resolved by acetone and filtered. The filtrate was evaporated by rotary evaporator, and purified by columned by using petroleum / ethyl acetate (3:1, v/v) to yield **1c** as yellow crystals (2.52 g, 38.5%). ^{1}H NMR (600 MHz, $CDCl_3$) δ 9.97 (s, 3H), 7.87 (d, *J* = 8.6 Hz, 6H), 7.27 (d, *J* = 8.5 Hz, 6H). ^{13}C NMR (151 MHz, $CDCl_3$) δ 190.48, 151.20, 132.60, 131.50, 124.54. MS (MALD-TOF): m/z: 328.369.

The synthesis of 2

2.3 g (7.64 mmol) of **1a** and 2.8 g (7.64 mmol) of 4,5-bis(hexylthio)-1,3-dithiole-2-thione were dissolved in 35 mL of boiling chlorobenzene, then 5 mL of triethylphosphite are added. The mixture is refluxed for 8 h under an argon atmosphere. After cooling, the mixture solution were evaporated by rotary evaporator,The residue is purified by chromtographed (CS_2/Triethylamine 95:5(v/v)) leading to a yellow oil ***2*** (1.29g, 28%). ^{1}H NMR (600 MHz, $CDCl_3$) δ 9.84 (s, 1H), 7.71 (d, *J* = 8.7 Hz, 2H), 7.37 (t, *J* = 7.9 Hz, 2H), 7.23 – 7.13 (m, 6H), 7.06 (d, *J* = 8.7 Hz, 2H), 6.46 (s, 1H), 2.85 (dt, *J* = 13.5, 6.7 Hz, 4H), 1.67 (dq, *J* = 15.2, 7.6 Hz, 4H), 1.44 (dq, *J* = 14.4, 7.3 Hz, 4H), 1.37 – 1.26 (m, 8H), 0.91 (dt, *J* = 11.5, 6.9 Hz, 6H). ^{13}C NMR (101 MHz, Acetone) δ 189.69(s), 152.90 (s), 146.11 (s), 143.84 (s), 133.04 (s), 131.22 (s), 130.99 (s), 129.87 (d, *J* = 9.3 Hz), 128.11 (s), 127.67 (s), 126.46 (s), 125.86 (s), 125.35 (s), 124.26 (s), 119.54 (s), 113.90 (s), 35.63 (d, *J* = 10.2 Hz), 31.22 (s), 29.67 (d, *J* = 16.8 Hz), 27.96 (d, *J* = 4.9 Hz), 22.38 (d, *J* = 1.2 Hz), 13.50 (s). MS (MALD-TOF): m/z: 619.582

The synthesis of 3

The component **3** was synthesized as the method as the **2**. **1b** (1.72g, 5.23mmol) and 4,5-bis(hexylthio)-1,3-dithiole-2-thione(5.74g, 15.69mmol) are dissolved in 35 mL of boiling chlorobenzene, then 6 mL of dry triethylphosphite are added. The mixture is refluxed for 8 h under an argon atmosphere. After cooling, the mixed solution was evaporated by rotary evaporator. The residue is purified by chromtographed using CH_2Cl_2/CS_2=3:7(v/v) to yield **3** as yellow oil (2.02 g, 40%). ^{1}H NMR (600 MHz, Acetone) δ 9.85 – 9.78 (m, 1H), 7.76 – 7.60 (m, 2H), 7.29 – 7.03 (m, 10H), 6.55 – 6.39 (m, 2H), 2.94 – 2.75 (m, 8H), 1.69 (t, *J* = 11.7 Hz, 8H), 1.50 (s, 8H), 1.43- 1.31 (m, 16H), 0.96 (tt, *J* = 19.2, 9.4 Hz, 12H). ^{13}C NMR (151 MHz, Acetone) δ 189.15, 152.44, 143.54, 133.23, 131.85, 131.06, 130.20, 128.23, 127.82, 125.90, 124.58, 120.28, 114.01, 36.08, 35.97, 31.59, 31.58, 30.07, 29.93, 28.40, 28.39, 22.83, 13.96, 13.94. MS (MALD-TOF): m/z: 966.538

The synthesis of 4

1100 mg (1.5 mmol) of C_{60}, 267 mg (3 mmol) of sarcosine, and 930 mg (1.5 mmol) of **2** were dissolved in toluene (35 mL). After heating at 120 ^oC and 4 hour, the reaction solution is cooled. The brown solution was concentrated and the raw solid product was purified by flash column chromatography on silica gel (200-300 mesh, CS_2 and toluene/hexane=1/2(v/v)) to give monocycloadduct product **4** (690mg, 34%). ^{1}H NMR (600 MHz, $CDCl_3$) δ 7.70 (s, 2H), 7.23 (t, *J* = 7.8 Hz, 2H), 7.14 (d, *J* = 5.9 Hz, 2H), 7.08 – 6.99 (m, 5H), 6.97 (d, *J* = 8.6 Hz, 2H), 6.39 (s, 1H), 5.08 – 4.78 (m, 2H), 4.28 (d, *J* = 9.2 Hz, 1H), 2.89 (s, 3H), 2.82 (dt, *J* = 16.3, 7.4 Hz, 4H), 1.65 (tt, *J* = 14.9, 7.5 Hz, 4H), 1.43 (dq, *J* = 14.2, 6.9 Hz, 4H), 1.35 – 1.27 (m, 8H), 0.91 (q, *J* = 7.1 Hz, 6H). ^{13}C NMR (151 MHz, $CDCl_3$) δ 156.30, 153.97, 153.65, 153.36, 147.72, 147.31, 147.22, 146.83, 146.50, 146.31, 146.29, 146.21, 146.17, 146.10, 145.94, 145.79, 145.55, 145.53, 145.47, 145.35, 145.30, 145.26, 145.21, 145.18, 145.15, 144.71, 144.69, 144.39, 144.36, 143.17, 143.00, 142.68, 142.62, 142.60, 142.57, 142.27, 142.25, 142.16, 142.13, 142.05, 141.79, 141.66, 140.17, 140.12, 139.91, 139.22, 136.67, 136.57, 135.93, 135.81, 131.60, 130.79, 130.18, 129.31, 127.69, 124.48, 124.30, 123.53, 123.10, 114.18, 83.03, 77.51, 70.01, 68.95, 40.12, 36.23, 36.05, 31.41, 31.37, 29.84, 29.69, 28.31, 22.61, 22.60, 14.14, 14.11. MS (MALD-TOF): m/z: 1369.11

The synthesis of 5

The synthesis of **5** is involved as same as **4.** 1100mg (1.5 mmol) of C_{60} , 267 mg (3.0 mmol) of sarcosine, and 1400mg (1.5 mmol) **3** were dissolved in toluene (35 mL). The mixed solution was fluxed and monitored by TLC. The reacted brown solution was concentrated and the raw solid product was purified by flash column chromatography on silica gel (100-200 mesh, CS_2 and toluene/hexane=1/1(v/v)) to give monoadduct product **5** (636mg, 31%). ^{1}H NMR (600 MHz, $CDCl_3$) δ 7.73 (d, *J* = 7.2 Hz, 2H), 7.15 (s, 2H), 7.07 (d, *J* = 8.6 Hz, 4H), 6.98 (d, *J* = 8.6 Hz, 4H), 6.39 (s, 2H), 4.98 (d, *J* = 27.3 Hz, 2H), 4.30 (d, *J* = 8.8 Hz, 1H), 2.91 (s, 3H), 2.87 – 2.75 (m, 8H), 1.65 (tt, *J* = 14.9, 7.5 Hz, 8H), 1.43 (dq, *J* = 14.3, 7.1 Hz, 8H), 1.37 – 1.26 (m, 16H), 0.91 (q, *J* = 7.1 Hz, 12H). 13C NMR (151 MHz, $CDCl_3$) δ 156.28, 153.62, 153.30, 147.43, 147.30, 146.80, 146.47, 146.32, 146.28, 146.20, 146.15, 146.09, 145.93, 145.77, 145.52, 145.46, 145.43, 145.33, 145.29, 145.25, 145.17, 145.14, 144.82, 144.70, 144.38, 144.34, 143.17, 142.98, 142.67, 142.61, 142.56, 142.26, 142.24, 142.15, 142.13, 142.07, 142.04, 142.03, 141.77, 141.66, 141.64, 140.16, 140.11, 139.90, 139.18, 136.66, 136.55, 135.94, 135.79, 132.08, 130.98, 130.32, 127.69, 124.49, 123.61, 114.08, 82.99, 77.53, 70.03, 68.94, 40.10, 36.22, 36.04, 31.40, 31.37, 29.83, 29.68, 28.30, 22.60, 22.59, 14.12, 14.09. MS (MALD-TOF): m/z: 1716.809

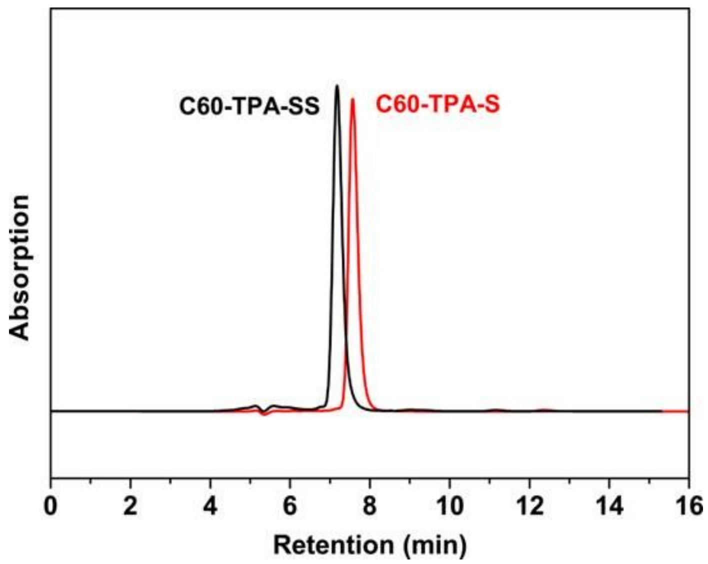

Figure S1. HPLC profiles of the obtained purified **4** (red) and **5** (black), on a Buckyprep 10 x 250 mm, chlorobenzene 0.6 mL/min, 330 nm detection wavelength.

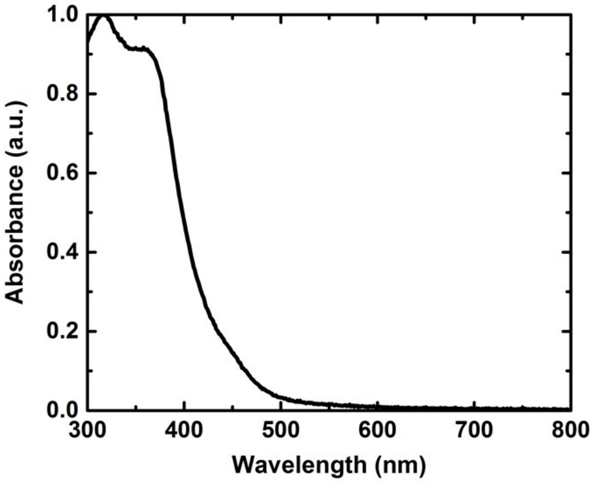

Figure S2. Normalized absorption spectrum of **3** in toluene.

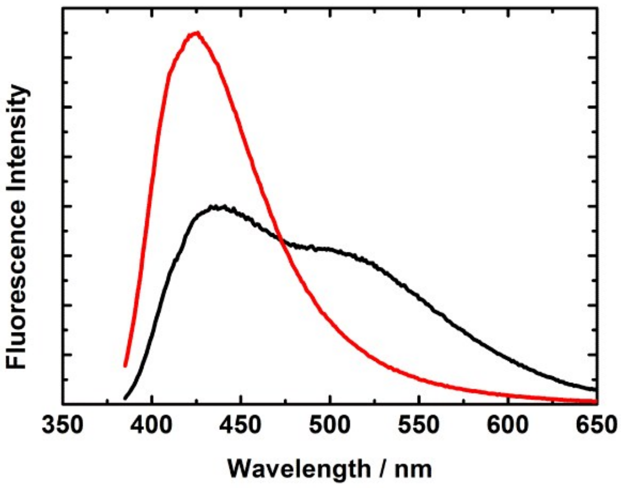

Figure S3. Fluorescence spectra of **2** (black) and **3** (red) in toluene upon photoexcitation at 370 nm.

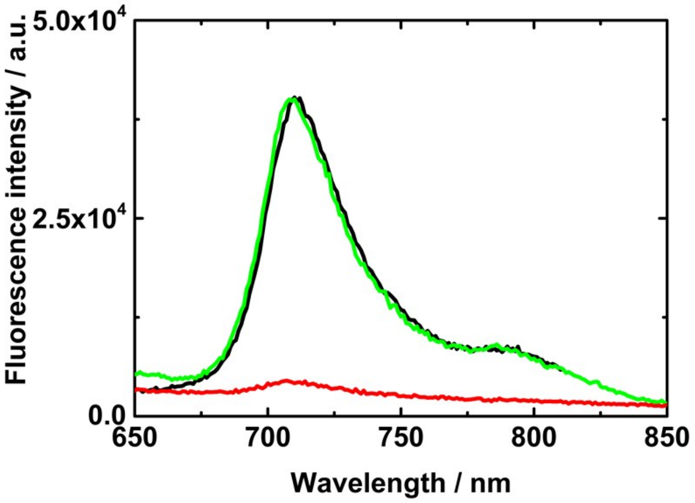

Figure S4. Emission spectra of **4** (black, 7.7 x 10^{-5} M), **5** (green), and **6** (red) in toluene with concentrations of 6.2 x 10^{-5} M upon photoexcitation at 470 nm.

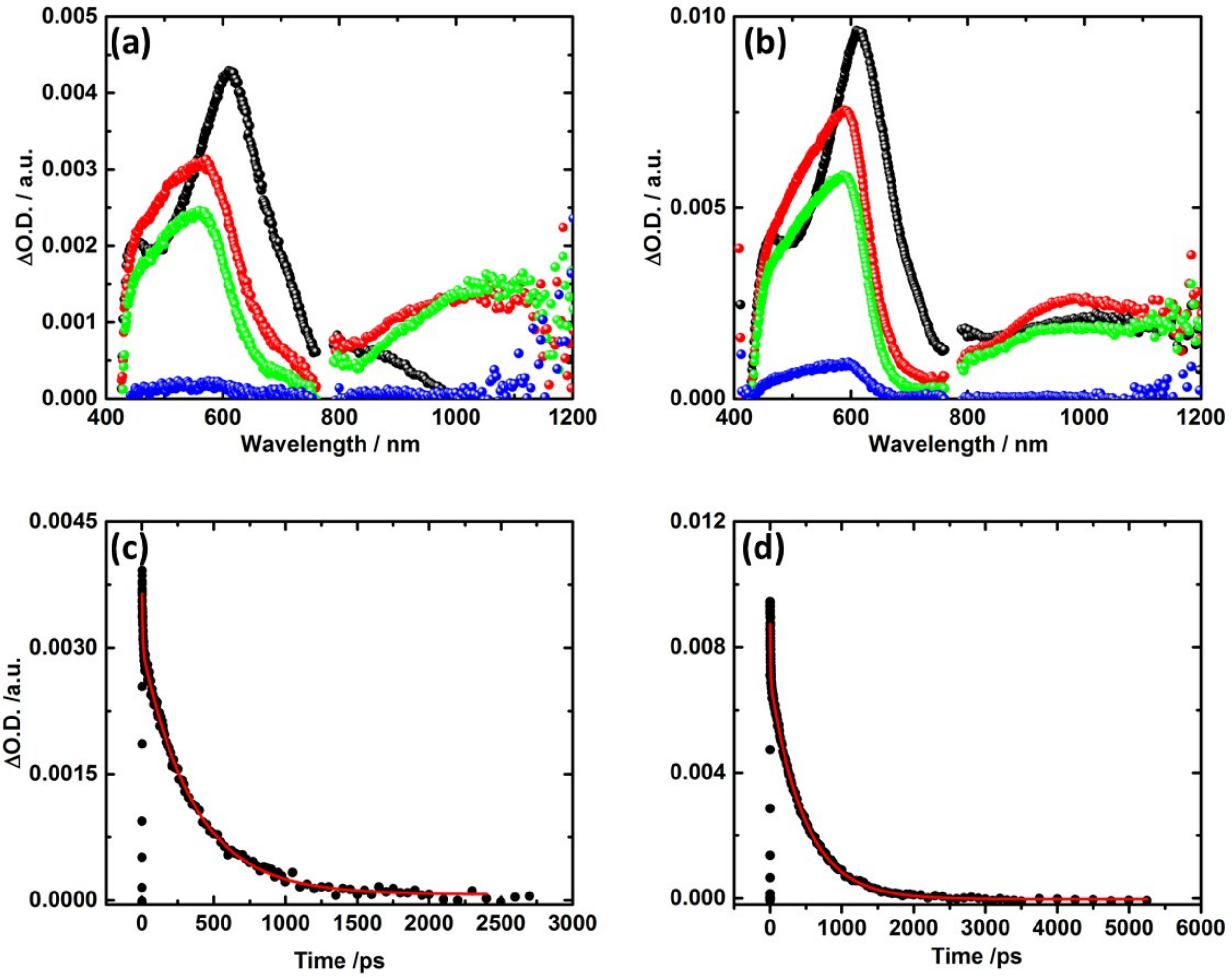

Figure S5. (**a**) Transient absorption spectra of **2**, and (**b**) **3** in argon saturated THF upon fs-laser photolysis (387 nm, 400 nJ / pulse) with time delays of 2 ps (black), 10 ps (red), 100 ps (green) and 1000 ps (blue), (**c**) corresponding time absorption profiles (black) and exponential fit (red) of **2** at 577 nm , and (**d**) time absorption profile (black) and exponential fit (red) of **3** at 598 nm.

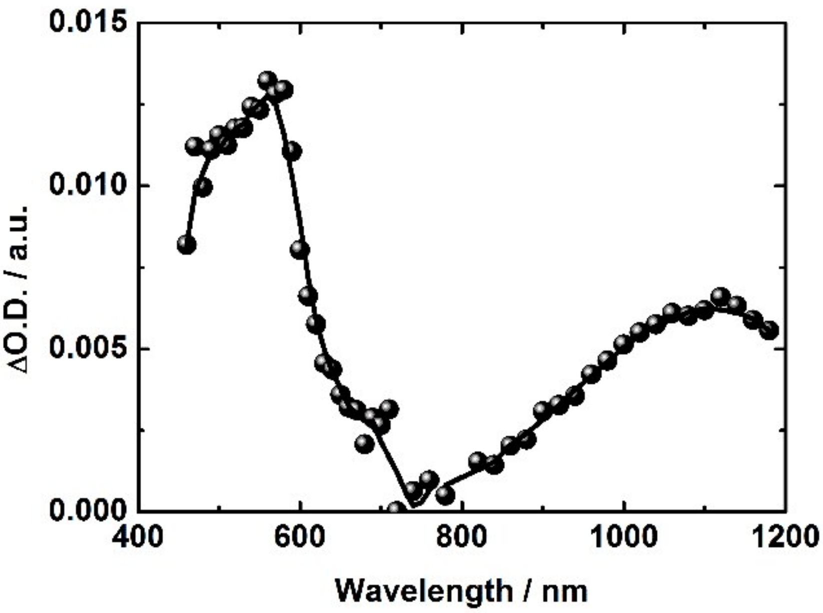

Figure S6. Pulse radiolysis spectrum of **3** (1×10^{-3} M) in N_2 saturated BuCl with a time delay of 100 ns after the electron pulse (100 Gy, 15 ns fwhm).

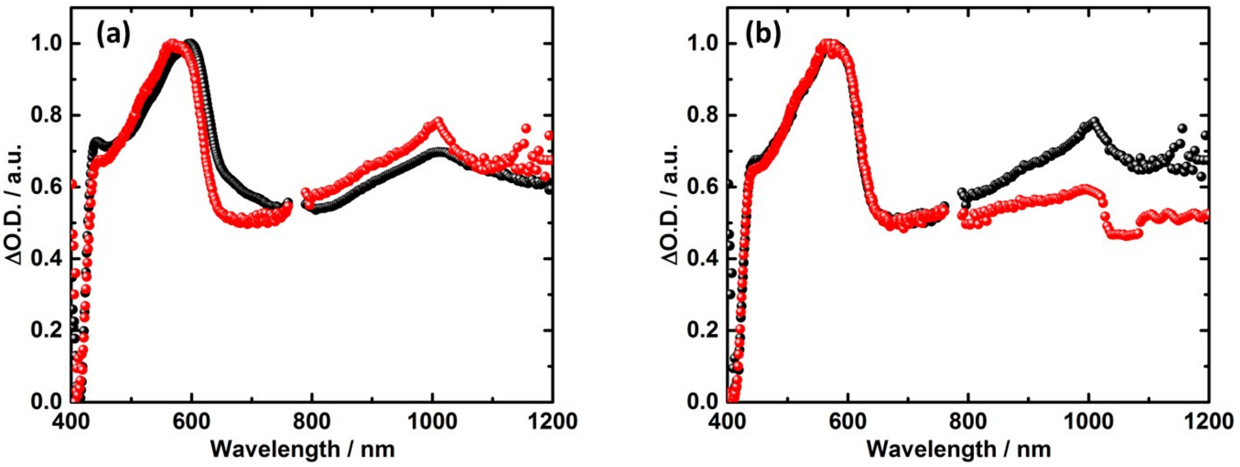

Figure S7. (**a**) Normalized transient absorption spectra of **5** in argon saturated THF upon fs-laser photolysis (387 nm, 400 nJ / pulse) with time delays of 2 ps (black), and 3000 ps (red) after the laser pulse (**b**) normalized transient absorption spectra (visible) of **5** in argon saturated THF upon fs-laser photolysis (387 nm, 400 nJ / pulse) with time delays of 3000 ps (black), and nanosecond transient absorption spectra with time delays of 3 ns (red) after the laser pulse.

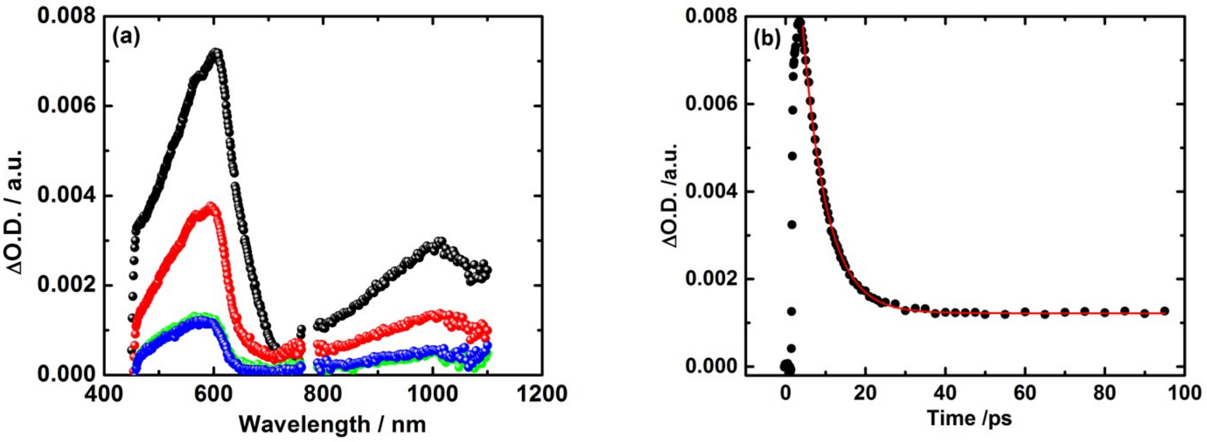

Figure S8. (**a**) Transient absorption spectra of **5** in argon saturated THF upon fs-laser photolysis (420 nm, 400 nJ / pulse) with time delays of 2 ps (black), 10 ps (red), 100 ps (green), and 1000 ps (blue) after the laser pulse (**b**) corresponding time absorption profile (black) and exponential fit (red) at 590 nm.

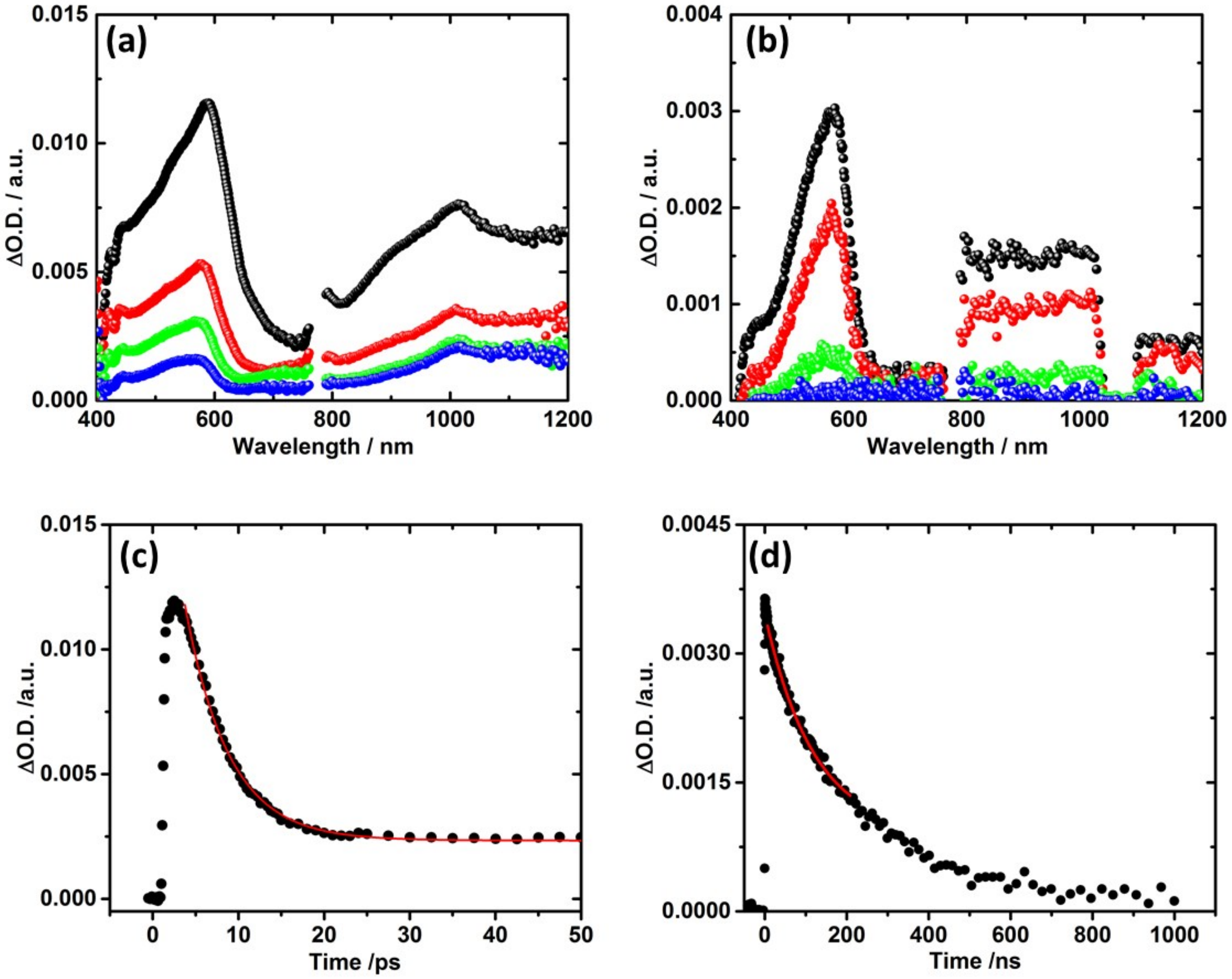

Figure S9. (**a**) Transient absorption spectra of **4** in argon saturated THF upon fs-laser photolysis (387 nm, 400 nJ / pulse) with time delays of 2 ps (black), 10 ps (red), 100 ps (green) and 5000 ps (blue) (**b**) 5 ns (black), 100 ns (red), 500 ns (green), and 1000 ns (blue), (**c**) corresponding time absorption profiles at 590 nm for shorter time scale (black) and exponential fit (red), (**d**) time absorption profile at 570 nm for longer timescale (black) and exponential fit (red).

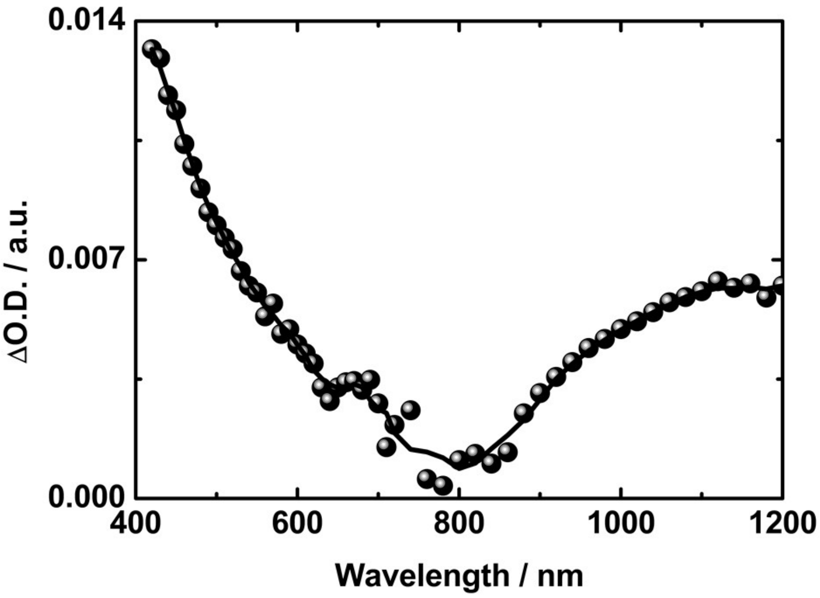

Figure S10. Pulse radiolysis spectrum of **2** (5×10^{-4} M) in N_2 saturated BuCl with a time delay of 100 ns after the electron pulse (100 Gy, 15 ns fwhm).

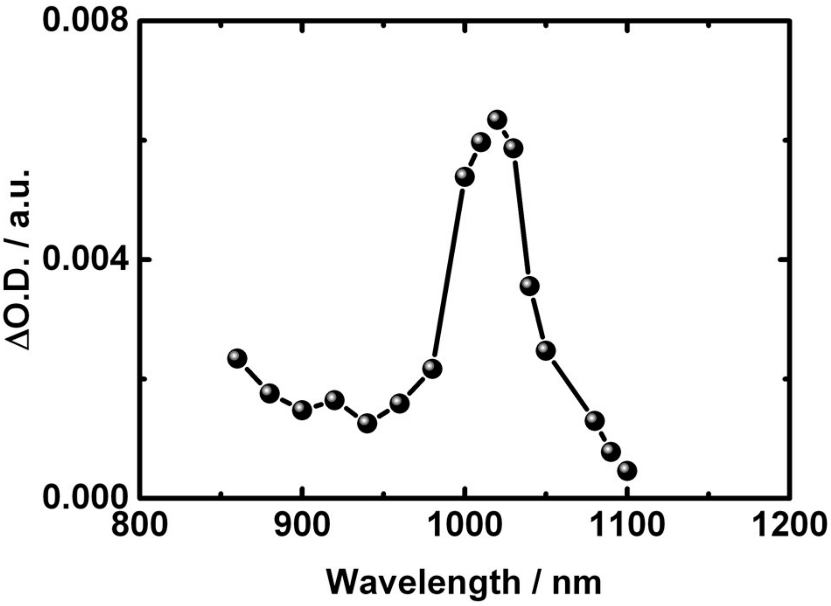

Figure S11. Nanosecond transient absorption spectra (NIR region) of **4** in argon saturated THF upon laser photolysis (355 nm, 10 mJ / pulse) with time delays of 50 ns after the laser pulse.

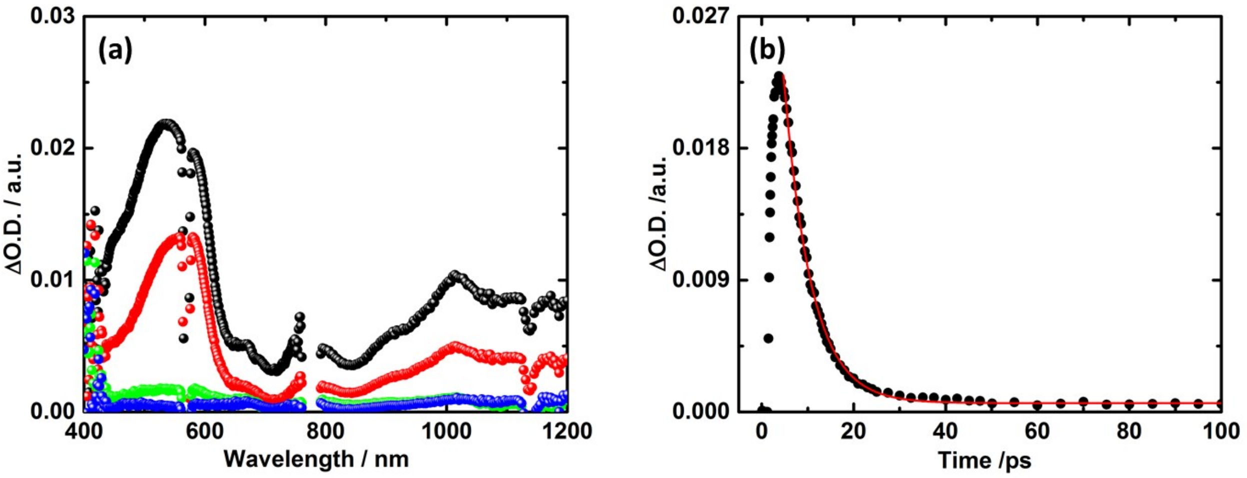

Figure S12. (**a**) Transient absorption spectra of **4** in argon saturated THF upon fs-laser photolysis (568 nm, 500 nJ / pulse) with time delays of 2 ps (black), 10 ps (red), 25 ps (green), and 100 ps (blue) after the laser pulse (**b**) corresponding time absorption profiles (black) and exponential decay fit (red) at 600 nm.
